# Towards Bioethical and Functional Standards in the Slaughter Methods of Edible Insects: A Narrative Review

**DOI:** 10.3390/insects17040424

**Published:** 2026-04-16

**Authors:** Oscar Abel Sánchez-Velázquez, Alan Javier Hernández-Álvarez

**Affiliations:** 1 School of Food Science & Nutrition, University of Leeds, Leeds LS2 9JT, UK; a.j.hernandezalvarez@leeds.ac.uk; 2 National Alternative Protein Innovation Centre (NAPIC), Leeds LS2 9JT, UK

**Keywords:** entomophagy, bioethics, insect welfare, insect farming, nociception, nutritional quality, techno-functional properties, bioactive compounds, insect killing methods

## Abstract

The rapid expansion of edible insect production has focused primarily on rearing, processing efficiency, safety, and nutritional composition, while the slaughter of insects has received comparatively little scientific and ethical scrutiny. This narrative review examines insect slaughter as a critical control point linking bioethics, physiology, and ingredient quality. The review synthesizes evidence from neurobiology, food science, and processing studies to evaluate how commonly used slaughter methods interact with biological aspects of insects. Existing literature shows that slaughter techniques influence protein stability and hydrolysis, lipid oxidation, antioxidant retention, techno-functional properties such as emulsification and gelation, as well as sensory attributes and consumer acceptance. Available evidence suggests that methods designed to rapidly suppress metabolic activity may be associated with improved preservation of certain nutritional and functional parameters, although findings remain species- and context-dependent. The review further highlights major knowledge gaps, including the lack of species- and life-stage-specific welfare indicators and standardized assessment protocols. Overall, the findings support the need to reconceptualize insect slaughter as a strategic upstream decision rather than a neutral processing step. Integrating ethical considerations with nutritional, functional, and regulatory perspectives is essential for establishing science-based standards and ensuring the responsible development of edible insect-based food and feed systems.

## Simple Summary

Edible insects are increasingly recognized as a sustainable source of food and feed. However, the methods used to kill insects before processing have received relatively little attention compared with traditional livestock. These methods can influence not only ethical considerations related to animal welfare but also the nutritional quality and technological properties of insect-derived ingredients. This review examines current slaughter practices used for edible insects and evaluates their potential implications for welfare, nutrient stability, and ingredient functionality. The available evidence shows that different killing methods can affect biochemical processes such as lipid oxidation, protein stability, and the preservation of bioactive compounds. Importantly, these effects are not determined by slaughter conditions alone but also by subsequent stabilization steps such as blanching, drying, freezing, or grinding. We therefore propose that insect processing should be considered within a broader slaughter-to-stabilization continuum in which early processing decisions influence final ingredient quality. The review also highlights significant knowledge gaps, including the limited number of species and developmental stages that have been studied. Developing species-specific slaughter protocols and welfare indicators will be essential for ensuring ethical, safe, and high-quality insect production systems.

## 1. Introduction

The accelerating demand for sustainable protein sources has intensified global interest in edible insects for both human food and animal feed applications. Insects are widely recognized for their favorable feed conversion efficiency, low greenhouse gas emissions, reduced land and water requirements, and high nutritional density when compared to conventional livestock [1,2,3]. Currently, more than two billion people consume insects as part of their traditional diets, particularly across Africa, Asia, and Latin America, while Western countries have witnessed a rapid expansion of industrial insect farming supported by emerging regulatory frameworks, such as Regulation EU 2015/2283 and UK FSA [1].

As the edible insect sector continues to scale up, research and regulation have largely focused on production efficiency, food safety, allergenicity, and nutritional composition [4,5]. In contrast, the slaughter of insects—defined as the process by which insects are intentionally killed prior to processing or consumption—remains strikingly underexplored from ethical, nutritional, and technological perspectives [6,7].

Historically, insects have been excluded from animal welfare discourse based on the assumption that they lack the neurological complexity required to experience pain or suffering [8]. Consequently, insect slaughter has often been treated as a purely technical pre-treatment step rather than as a biologically and ethically meaningful process. However, this position has been increasingly challenged by advances in neuroethology and behavioral science, which suggest that several insect taxa exhibit nociceptive responses, learning from aversive stimuli, and long-term behavioral alterations following injury [9,10]. Although definitive evidence of subjective pain experience in insects remains elusive, these findings raise legitimate ethical questions regarding how insects are killed at industrial scales.

Beyond ethics, slaughter methods exert a profound influence on the nutritional, bioactive, sensory, and techno-functional properties of insect-derived ingredients [11]. A growing body of literature demonstrates that killing techniques such as freezing, boiling, CO_2_ exposure, mechanical crushing, or direct thermal drying can differentially affect protein integrity, lipid oxidation, vitamin stability, bioactive compound retention, and functional performance in food and feed formulations [7,11,12,13,14]. Despite this evidence, slaughter is still rarely considered a controllable variable for ingredient optimization, with downstream processing steps (e.g., drying, defatting, protein extraction) receiving disproportionate attention [15]. Importantly, the consequences of insect-killing methods extend beyond immediate welfare considerations. The biochemical stability, nutritional quality, and techno-functional properties of insect-derived ingredients are shaped by the interaction between killing procedures and subsequent stabilization processes such as blanching, drying, freezing, or grinding. Therefore, insect slaughter should be understood as the first stage of a broader slaughter-to-stabilization continuum, in which early processing decisions influence downstream ingredient quality and product performance.

An additional layer of complexity arises from the life stage at which insects are slaughtered. Developmental stage determines not only compositional attributes such as lipid content, chitin levels, and protein profile, but also neurological development, metabolic activity, and stress responsiveness [16,17]. Eggs, larvae, pupae, and adults may therefore differ substantially in both their potential welfare vulnerability and the quality of the ingredients they yield, yet life-stage-specific slaughter considerations remain poorly integrated into current production systems [17,18].

Against this backdrop, the present narrative review critically examines insect slaughter as a multidimensional process at the intersection of bioethics, biology, and food science. Specifically, this review aims to: (i) synthesize current evidence on insect nociception and ethical uncertainty; (ii) evaluate commonly used slaughter methods through physiological, ethical, and technological lenses; (iii) analyze how slaughter methods and life stage interact to shape nutritional, bioactive, sensory, and techno-functional outcomes; (iv) propose a conceptual foundation for the development of ethical, scientifically grounded slaughter standards for edible insects.

## 2. Methodological Approach and Scope of the Narrative Review

This work adopts a narrative review approach, selected to integrate and critically interpret evidence across multiple disciplines, including neuroethology, animal ethics, food science, nutrition, and insect production systems. Given the conceptual and methodological heterogeneity of the available literature, a systematic review or meta-analysis was deemed unsuitable for addressing the multifaceted research questions posed in this study.

A comprehensive literature search was conducted between April and December 2025 using the databases Scopus (https://www.scopus.com/), Web of Science (https://www.webofscience.com/), and Google Scholar (https://scholar.google.com/). In addition, technical and regulatory documents from international organizations, including the FAO [1,19], EFSA [20,21], and other relevant policy bodies, were consulted. Search terms included combinations of “edible insects,” “slaughter methods,” “killing methods,” “euthanasia,” “insect welfare,” “nociception,” “bioethics,” “techno-functional properties,” “bioactive compounds,” and scientific name of insect species (such as “*Locusta migratoria*,” “*Acheta domesticus*,” “*Hermetia illucens*,” “*Tenebrio molitor*,” “*Zophobas morio*,” “*Bombyx mori*,” “*Drosophila melanogaster*,” “*Alphitobius diaperinus*,” “*Gryllus sigillatus*,” among others). Although a wide diversity of edible insect species was considered during the literature search, only a limited number of species and developmental stages were represented in studies evaluating slaughter methods and their effects on nutritional, biochemical, or techno-functional properties. This uneven distribution of evidence reflects a broader knowledge gap in the field, where research remains strongly concentrated on a few commercially farmed species, while many other edible insects and life stages remain largely unexplored. Studies were screened based on their relevance to insect killing methods and their potential impacts on nutritional composition, bioactive compounds, and techno-functional properties of insect-derived ingredients.

The inclusion criteria comprised: (a) peer-reviewed articles published between 2010 and 2025; (b) studies addressing insect slaughter, killing, or pre-processing conditions; (c) research examining ethical, neurological, nutritional, functional, bioactive, or sensory outcomes of insect processing; and (d) empirical studies, theses, and authoritative technical reports providing original data. During the screening stage, duplicate records were removed and titles and abstracts were evaluated for relevance to the objectives of this review. Excluded records were categorized into three main groups: (i) duplicate entries identified during database merging; (ii) studies with limited relevance to insect slaughter or killing methods (e.g., publications focused primarily on insect nutrition, rearing systems, ecology, or general processing without reference to slaughter practices); and (iii) publications addressing invertebrate taxa outside the scope of this review, including cephalopods, mollusks, decapod crustaceans, and other aquatic arthropods.

From an initial pool of 253 documents, 68 references were retained following relevance screening and critical appraisal. Rather than quantitatively aggregating results, the selected literature was analyzed thematically, emphasizing points of convergence and divergence, methodological limitations, species-specific and life-stage-specific gaps, and the frequent detachment between welfare considerations and ingredient quality assessment. This process also revealed that the available literature remains strongly concentrated on a limited number of commercially farmed insect species, highlighting an important gap in the evidence base for many edible insects. This narrative synthesis intentionally prioritizes conceptual integration over exhaustiveness, aiming to construct an evidence-informed framework that can guide future experimental design, industrial practice, and regulatory development in the slaughter of edible insects.

## 3. Ethical Foundations: Pain, Sentience, and the Precautionary Principle

Determining whether insects are capable of experiencing pain is a central consideration in many ethical evaluations of their slaughter for food and feed [6,16,22,23]. However, moral concern about insect slaughter does not necessarily depend solely on evidence of pain perception. Several frameworks in animal welfare science emphasize alternative criteria, such as biological functioning or the ability of animals to cope with environmental challenges [24,25,26]. From these perspectives, practices that cause severe physiological disruption or prevent organisms from responding adaptively to their environment may raise welfare concerns even in the absence of direct evidence of subjective pain. In addition, some ethical and religious traditions extend moral consideration to living organisms independently of their capacity for sentience. For example, Jain philosophical traditions emphasize the principle of ahimsa (non-violence) toward all living beings [27], while certain Buddhist perspectives similarly encourage minimizing harm to living organisms even when their capacity for suffering is uncertain [28,29]. These perspectives illustrate that ethical concern about insect slaughter may arise from multiple normative frameworks, not exclusively from the question of pain perception.

Traditionally, insects were excluded from moral consideration on the basis that their nervous systems lack the centralized structures—such as a neocortex—associated with conscious pain perception in vertebrates [9]. Early work by Eisemann et al. [8] argued that insect responses to harmful stimuli were best understood as nociceptive reflexes rather than indicators of subjective suffering. Over the past two decades, this view has been increasingly challenged by accumulating evidence from behavioral, neurobiological, and theoretical studies examining the possibility of pain-like states in insects [30]. Advances in neuroethology and behavioral ecology have revealed that several insect species exhibit responses to noxious stimuli that extend beyond simple reflex arcs [22]. These include long-term avoidance learning, site-specific grooming, reduced locomotion, altered foraging strategies, and motivational trade-offs following injury [9,10,31]. Such responses have been documented in taxa directly relevant to entomoculture, including *Drosophila* spp. and *Acheta domesticus* [32,33,34].

Importantly, the absence of a neocortex is no longer considered definitive evidence against the possibility of pain. Klein and Barron [35] proposed that affective states may arise from simpler neural architectures through functional equivalence, provided that organisms can integrate sensory information, learn from aversive experiences, and flexibly modify behavior. Insects have repeatedly demonstrated such capacities. For example, *Drosophila* larvae and adults display sensitization following injury, prolonged hypervigilance, and modulation of nociceptive responses by pharmacological agents, including opioids [36,37,38]. Despite these findings, it remains scientifically unresolved whether insect nociception corresponds to a subjective experience of pain. Insects cannot communicate affective states verbally, and their neural organization differs fundamentally from that of vertebrates [35,39]. This epistemic uncertainty does not eliminate ethical responsibility. Instead, it strengthens the relevance of the precautionary principle, which holds that when credible evidence of potential harm exists, the absence of full scientific certainty should not be used to justify inaction [40].

Comparable reasoning has already informed regulatory developments in other invertebrate groups [41]. In Europe, accumulating evidence of sentience in cephalopods and decapod crustaceans led to their inclusion in animal welfare legislation [42]. Similarly, the European Food Safety Authority has acknowledged the current uncertainty regarding insect sentience and has called for further research to inform ethical and regulatory decisions [20]. These developments suggest that insects may eventually be brought into the scope of welfare considerations as scientific understanding evolves.

The ethical implications are particularly acute in industrial insect farming, where millions to billions of individuals are killed daily using methods selected primarily for efficiency and cost [43]. Under a precautionary framework, the burden of justification shifts toward producers and regulators, who must demonstrate that slaughter methods plausibly minimize suffering rather than assuming its absence [41].

Significant gaps remain in the literature. Most experimental studies focus on a narrow set of model organisms, such as *Drosophila* spp. [38,44,45,46,47], with limited data on commercially relevant species including *Hermetia illucens*, *Tenebrio molitor*, *Alphitobius diaperinus*, or *Gryllus sigillatus* [41]. Moreover, few studies combine behavioral assays with neurophysiological measurements, making it difficult to distinguish reflexive responses from centrally mediated experiences. Expanding research under standardized experimental frameworks to include a wider range of taxa relevant to insect farming—particularly beetles, flies, orthopterans, and cockroaches—represents a critical next step [31,48,49]. Although definitive proof of insect pain remains elusive, existing behavioral and neurobiological evidence justifies considering precautionary welfare measures. Slaughter methods that reduce stress, avoid extreme thermal shock, or incorporate anesthetic or stunning phases may therefore constitute an ethical advancement in entomoculture. Therefore, although the possibility of pain perception remains a central scientific and ethical question, broader welfare and moral frameworks may also justify concern regarding insect slaughter practices.

## 4. Slaughter Methods in Edible Insects: Physiological and Ethical Implications

A wide range of slaughter methods is currently employed in the production of edible insects, differing substantially in technological complexity, scalability, energy demand, ethical acceptability, and downstream effects on ingredient quality [23,50]. In contrast to vertebrate livestock, insects remain largely excluded from binding animal welfare legislation, resulting in heterogeneous, poorly standardized, and often undocumented killing practices across research, food, and feed sectors.

This regulatory vacuum has fostered an implicit assumption that slaughter methods for insects are ethically neutral or biologically inconsequential. However, accumulating evidence from neurobiology, physiology, and behavior challenges this view. Slaughter represents a biologically and ethically relevant step within industrial production systems.

**Figure 1:**
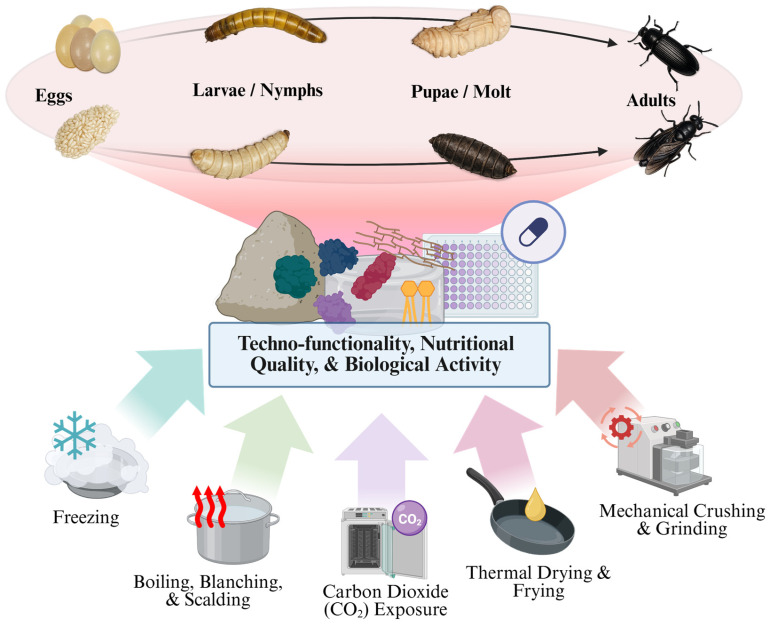
Conceptual framework illustrating how targeted techno-functional, nutritional, and bioactive properties of derived insect ingredients influence the selection of slaughter methods based on insect species and biological life stage.

The selection of insect slaughter methods is not determined by a single factor but emerges from the interaction between biological constraints (species and life-stage), and ingredient quality objectives (nutritional quality and bioactivities), as well as technological feasibility, and regulatory and consumer contexts (Figure 1). This section critically evaluates the most commonly used slaughter techniques through integrated physiological and ethical lenses, emphasizing uncertainty, precaution, and species-specific variability.

### 4.1. Freezing

Freezing is among the most widely adopted slaughter methods in both experimental and commercial insect farming. In practice, insects are exposed to decreasing temperatures—commonly ranging from refrigeration (~4 °C) to deep freezing (−20 to −80 °C)—inducing progressive torpor, metabolic suppression, and eventual death [51,52]. Freezing of insects may be achieved through conventional mechanical freezing (e.g., −20 to −80 °C freezers) or via rapid cryogenic freezing using liquid nitrogen [53,54]. While both approaches result in death through cold-induced metabolic and neural suppression, their markedly different cooling rates raise distinct ethical and physiological considerations regarding the timing of loss of consciousness and potential nociceptive activation [55,56]. From a physiological standpoint, cold exposure reduces neuronal firing rates, muscular activity, and oxygen consumption, potentially limiting the capacity for nociceptive processing if cooling occurs sufficiently gradually.

Despite its widespread use in insect farming, freezing remains controversial from an animal welfare perspective. Several veterinary and animal welfare guidelines do not currently recognize freezing as a humane euthanasia method for invertebrates. The American Veterinary Medical Association (AVMA) guidelines for the euthanasia of animals note that slow cooling or direct freezing may cause prolonged neural activity prior to loss of consciousness and therefore cannot be assumed to minimize distress [57]. Similarly, guidance developed for zoological institutions and research facilities discourages freezing as a primary euthanasia method for terrestrial invertebrates [58,59]. These guidelines emphasize that insufficient empirical data currently exist to demonstrate that freezing results in rapid insensibility in insects.

Recent syntheses of invertebrate euthanasia practices have similarly highlighted the lack of robust empirical evidence supporting freezing as a welfare-compatible killing method. For example, Bakker et al. [60] concluded that although freezing is widely applied in research and industry due to its practicality, current data remain insufficient to determine whether it reliably prevents nociceptive responses or distress across insect taxa.

This physiological rationale has contributed to the perception among some insect producers that freezing may represent a relatively humane method, although this assumption remains debated in the veterinary and animal welfare literature. Qualitative studies among UK insect farmers indicate that freezing is often chosen explicitly for moral reasons, as it is believed to mimic natural nocturnal or seasonal cooling and to minimize suffering while aligning with consumer expectations of welfare-friendly practices [41]. Importantly, this ethical justification is largely inferential rather than evidence-based.

Critical uncertainties remain regarding the rate of cooling, species-specific cold tolerance, and life-stage-dependent neural shutdown [55,56]. Rapid freezing may induce cold shock before loss of consciousness, whereas slow cooling may prolong exposure to sublethal temperatures during which nociceptive processing could persist [61]. Insects adapted to cold environments or capable of chill tolerance may remain physiologically responsive at temperatures assumed to induce unconsciousness in other species [62]. From an ethical perspective, these uncertainties complicate claims of humaneness and highlight the need for species-specific validation rather than generalized assumptions. In addition to welfare considerations, freezing may also influence post-mortem biochemical stability. For example, Caligiani et al. [63] reported that freezing of *H. illucens* prepupae promoted extensive lipolysis during storage due to continued lipase activity, whereas blanching rapidly inactivated these enzymes and preserved lipid integrity.

From a regulatory perspective, current veterinary and animal welfare guidance does not clearly recognize freezing as a humane euthanasia method for terrestrial invertebrates. The acceptance of freezing as a welfare-compatible slaughter method would likely require stronger empirical evidence demonstrating rapid loss of neural function and the absence of prolonged nociceptive activity across insect taxa. In the absence of such data, veterinary authorities remain cautious about endorsing freezing as a humane approach. Consequently, future research should prioritize experimental evaluation of cooling rates, neural activity, behavioral indicators of distress, and species-specific responses in order to determine whether freezing can meet welfare criteria comparable to those applied in vertebrate slaughter systems.

### 4.2. Boiling, Blanching, and Scalding

Boiling, blanching, and scalding are closely related thermal slaughter methods that differ in the intensity and duration of heat exposure [41]. Boiling entails sustained immersion in water at or near 100 °C; blanching involves brief exposure to boiling water followed by rapid cooling; while scalding applies hot water or steam at sub-boiling temperatures for variable durations. From both physiological and ethical perspectives, these differences are highly relevant, as shorter or less intense thermal exposure may reduce—but not eliminate—the window for nociceptive activation prior to loss of neural function [31,46].

Thermal immersion methods such as boiling or blanching are frequently used in the preparation of insects for direct human consumption and for reduction in microbial load [41,64]. In industrial insect processing, blanching is commonly used not only as a killing step but also to reduce microbial loads and inactivate endogenous enzymes responsible for degradation reactions, including browning and lipid oxidation [65].

Sudden exposure to high temperatures is likely to elicit acute nociceptive responses in awake insects, particularly in species with demonstrated sensitivity to thermal stimuli such as *A. domesticus* and *Drosophila melanogaster* [10,66]. However, thermal nociception is not limited to these model organisms. Behavioral responses to lethal heat stress have also been documented in farmed species such as the black soldier fly (*H. illucens*), where larvae actively escape from high-temperature conditions in production environments [67]. In addition, comparative genomic analyses have shown that the transient receptor potential (TRP) channels associated with heat nociception in *D. melanogaster* are conserved across multiple insect taxa, including silkworms, black soldier flies, crickets, and yellow mealworms [68]. These findings suggest that sensitivity to noxious heat may be widespread among insects used in food and feed production.

Ethically, boiling or blanching without prior stunning may raise welfare concerns if insects remain responsive during the heating process. Even if death occurs quickly, the intensity of the stimulus and the likelihood of neural processing of noxious thermal stimuli challenge justifications based solely on speed. However, the ethical evaluation of thermal immersion should not rely solely on analogies with vertebrate slaughter practices.

Instead, the welfare implications of boiling depend primarily on how rapidly the method induces loss of neural function in the organism being processed. Methods that fail to produce rapid insensibility may cause prolonged nociceptive stimulation, whereas procedures that result in near-instantaneous death may present different welfare considerations. Because insects are small-bodied organisms with distinct physiological and thermal properties, the time required for lethal heat exposure may differ substantially from that observed in vertebrate animals. Consequently, the ethical evaluation of boiling should focus on species-specific physiological responses and validated operational parameters rather than on the method label itself.

Pre-stunning methods such as CO_2_ exposure or chilling are frequently proposed as mitigation strategies, yet their application remains inconsistent, poorly standardized, and rarely monitored. The ethical adequacy of thermal immersion, therefore, depends not only on the method itself but on the broader slaughter protocol within which it is embedded [41].

### 4.3. Carbon Dioxide (CO_2_) Exposure

CO_2_ exposure is widely used in entomological research as an anesthetic and has been proposed as a pre-slaughter stunning method in industrial insect production [69]. Elevated CO_2_ concentrations induce respiratory acidosis, suppress central nervous activity, and rapidly immobilize insects, potentially rendering them unconscious prior to killing. Physiologically, CO_2_ stunning offers several advantages: it is non-thermal, scalable, and can reduce stress-induced oxidative damage and metabolic disruption, as demonstrated in crickets subjected to CO_2_ prior to heat treatment [14]. These features position CO_2_ as a promising compromise between welfare and industrial feasibility. Ethical concerns have also been raised regarding the possibility of distress associated with hypercapnia and suffocation-like states, paralleling debates in vertebrate slaughter. Guidance from laboratory animal anesthesia literature similarly reflects a cautious position: although CO_2_ is widely used in insect research due to its practicality, it is not necessarily regarded as the most progressive anesthetic option for invertebrates, while still remaining acceptable in certain experimental contexts [69,70].

Sensitivity to CO_2_ varies markedly across species and developmental stages, and incomplete exposure may allow recovery. Moreover, hypercapnia may itself be aversive, potentially inducing panic-like or suffocation-related distress prior to loss of consciousness, paralleling long-standing ethical debates in vertebrate slaughter systems [71]. Without objective indicators of unconsciousness, it remains unclear whether CO_2_ exposure reliably minimizes suffering across taxa. Thus, while CO_2_ therefore represents a promising, stunning method within integrated slaughter protocols, its ethical acceptability cannot be assumed a priori and requires rigorous, species-specific validation.

### 4.4. Thermal Drying and Frying

In some industrial contexts, live insects are subjected directly to thermal drying without prior stunning. Although highly efficient and scalable, gradual thermal drying raises important welfare concerns in insect processing systems. Unlike rapid killing methods, it exposes insects to prolonged sublethal temperatures during which metabolic activity, neural processing, and stress responses may persist [7,41]. This extended exposure may increase the duration of nociceptive stimulation while offering no clear physiological mechanism for the rapid loss of neural function [8,31]. Consequently, gradual heating approaches warrant careful ethical scrutiny, particularly when alternative methods capable of inducing faster mortality are available.

On the other hand, frying, typically used in culinary contexts, involves immersion in hot oil and results in rapid death [50]. Nevertheless, as with boiling, immersion in hot oil may expose insects to extremely high temperatures that could trigger nociceptive pathways prior to loss of neural function. However, the extent to which such activation occurs in practice remains uncertain, particularly in small-bodied insects where lethal temperatures may be reached very rapidly. At sufficiently high temperatures, proteins involved in thermal nociception, including TRP channels, may rapidly denature, potentially limiting the transmission of nociceptive signals [68]. Consequently, thermal immersion methods such as boiling or frying cannot be dismissed solely on conceptual grounds; their welfare implications likely depend on the speed with which lethal temperatures are reached in a given species. Further empirical research is needed to determine whether these methods reliably induce rapid mortality without prolonged nociceptive processing.

### 4.5. Mechanical Crushing and Grinding

Mechanical crushing and grinding are predominantly used in animal feed production due to their speed, low cost, and compatibility with existing processing infrastructure. These methods often involve processing large numbers of live insects without stunning, raising significant welfare concerns.

Recent empirical work has begun to quantify the conditions under which mechanical grinding may function as a rapid slaughter method in farmed insects. Studies on black soldier fly larvae (*H. illucens*) indicate that appropriately configured grinding or shredding systems can achieve near-instantaneous mortality in up to 99% of processed individuals [72], although a small proportion of *H. illucens* or *T. molitor* larvae may remain temporarily trapped within processing equipment and experience delayed mortality [73]. While this represents a potential welfare concern, available evidence suggests that such cases likely constitute a minority of processed individuals and are strongly influenced by equipment design and operational conditions.

Complementary work by Zacarias et al. [74] on yellow mealworm larvae (*Tenebrio molitor*) further demonstrated that mortality outcomes depend substantially on processing parameters. Using a particle plate hole diameter of 2.55 mm combined with the addition of filler at the end of processing runs, resulted in a 96.47% probability of instantaneous death, whereas larger plate sizes and the absence of filler reduced this probability to approximately 73%.

Together, these findings indicate that welfare outcomes in mechanical slaughter are not intrinsic to the method itself but depend critically on species-specific physiological characteristics, equipment configuration, and standardized operating procedures designed to ensure consistently rapid killing. From an ethical standpoint, failure to optimize these variables represents a preventable welfare risk rather than an unavoidable consequence of large-scale processing.

### 4.6. Integrated Considerations and Hybrid Slaughter Approaches

At present, no slaughter method has been conclusively demonstrated to be universally optimal for farmed insects, largely because relatively little research has been devoted to systematically developing and validating such approaches. Methods currently used in insect production each present distinct advantages and limitations. Freezing, for example, is widely perceived as a welfare-aligned approach due to its practicality and resemblance to natural cold-induced torpor; however, some veterinary and welfare guidelines caution that gradual cooling may still permit neural activity and potentially cause distress prior to death [52,55,57,58,59,60]. Thermal immersion methods such as boiling or frying can achieve rapid mortality in small-bodied insects, yet their welfare implications depend strongly on whether insects are stunned beforehand or processed using appropriate standard operating procedures that ensure near-instantaneous death. CO_2_ exposure is commonly used in entomological research as an anesthetic and may provide a practical stunning method prior to killing, although physiological responses can vary considerably across species and exposure conditions [69,70]. Mechanical approaches, including grinding or shredding, can achieve rapid mortality when properly configured but require careful equipment design and operational controls to minimize the risk of delayed death in a small proportion of individuals [73,74].

These limitations highlight the need to move beyond ad hoc decision-making toward standardized, evidence-based slaughter protocols that integrate physiological indicators, ethical considerations, and technological design. Rather than relying on single techniques, increasing attention is being given to integrated strategies that combine multiple interventions in order to balance welfare outcomes with microbial safety and ingredient quality [63,64,71].

In this context, hybrid slaughter approaches are emerging as a promising direction in insect processing systems. Such approaches may involve pre-stunning techniques—such as CO_2_ exposure or chilling—followed by rapid thermal treatments like blanching, which can simultaneously reduce potential distress while ensuring effective microbial stabilization. Similarly, some processing schemes combine killing methods with immediate stabilization steps such as drying, freezing, or grinding to limit enzymatic degradation and oxidative reactions [64,65]. These sequential or combined interventions illustrate that slaughter practices should not be viewed as isolated events but rather as components of integrated processing systems.

From this perspective, evaluating combinations of methods may offer more practical and scalable solutions for industrial insect production than relying on individual techniques alone. Hybrid strategies therefore reinforce the concept of an integrated slaughter–to–stabilization continuum, in which killing and stabilization steps interact to determine both welfare outcomes and the final nutritional, biochemical, and functional quality of insect-derived ingredients.

## 5. Life Stage at the Time of Slaughter: Development, Metabolism, and Ethical Relevance

The developmental stage at which insects are slaughtered plays a pivotal role in determining both ethical vulnerability and ingredient quality [17]. Insects undergo either holometabolous or hemimetabolous development, resulting in life stages—larvae or nymphs, pupae, and adults—that differ markedly in neurobiology, metabolism, and composition [75].

From a neuroethological perspective, larval and nymphal stages are often assumed to possess less differentiated nervous systems than adults, potentially implying a lower capacity for pain or suffering [76]. However, larval insects exhibit robust nociceptive responses and escape behaviors, indicating functional sensory processing even at early stages [77]. Ethical assumptions regarding reduced suffering in larvae, therefore, remain speculative.

Metabolically, larval stages often exhibit higher lipid content, lower chitin levels, and rapid growth rates compared with later developmental stages. These characteristics contribute to their widespread use in food and feed production systems, although large-scale farming systems may not always allow precise separation between closely related growth stages [78]. These traits not only influence nutritional value but also affect processing efficiency and functional performance [79,80,81]. For example, protein content of *T. molitor* larvae, pupae and adults has been estimated in 47.7, 53.1, and 60.2 g/100 g dry matter basis (dmb), respectively [82], or in *A. domesticus* nymphs and adults, this content varies from 15.4 and 20.5 g/100 g dmb, respectively [83]. Larvae are also less mobile and more tolerant of crowding, which may reduce pre-slaughter stress during handling.

The pupal stage represents a unique biological and ethical window in insect production systems. Pupae are typically characterized by the absence of feeding behavior, reduced locomotion, and extensive internal reorganization as larval tissues are remodeled into adult structures. During early pupation, these processes are accompanied by substantial neural remodeling, which may temporarily alter sensory processing and behavioral responsiveness [84]. Such physiological conditions have led some authors to suggest that pupae may represent a developmentally less vulnerable stage from a welfare perspective compared to actively feeding larvae or mobile adults. However, the degree to which sensory perception is reduced likely varies across the pupal period. As metamorphosis progresses, the nervous system becomes progressively reconstituted into its adult configuration, meaning that late-stage pupae may function neurobiologically as nearly fully formed adults that remain temporarily immobilized prior to emergence. Consequently, assumptions about reduced sensory capacity during pupation should be made cautiously and may depend strongly on the specific timing within the pupal developmental trajectory. Examples include the use of *H. illucens* pupae and *Bombyx mori* pupae, the latter traditionally harvested for silk production but increasingly explored for food applications. However, pupae may be less desirable compositionally depending on species and intended use [80,85].

Adult insects often display more complex behaviors and possess higher chitin content due to exoskeletal maturation, which may reduce digestibility [86]. Species such as beetles, bees, and grasshoppers show marked increases in structural proteins and cuticular material with age, influencing both nutritional and techno-functional outcomes [80]. Life stage also intersects with consumer perception. Larval forms are frequently perceived as less animal-like and may elicit lower ethical resistance among consumers unfamiliar with entomophagy [80,87]. These psychological factors further complicate decisions regarding optimal harvest timing. Lastly, harvesting decisions reflect trade-offs between welfare, quality, and economic efficiency [80]. An integrated approach that tailors slaughter methods to species and developmental stage—potentially incorporating pre-slaughter conditioning such as temperature modulation or fasting—offers a pathway toward ethically and functionally optimized insect production systems.

## 6. Nutritional Impact of Slaughter Methods

The nutritional value of edible insects is a primary driver of their adoption as alternative protein sources for both human food and animal feed [19]. Insects provide high-quality proteins, essential amino acids, unsaturated fatty acids, vitamins, and minerals, positioning them as nutritionally competitive with conventional livestock [2,60]. However, accumulating evidence demonstrates that slaughter methods constitute a critical determinant of nutrient integrity, influencing both absolute nutrient content and bioavailability [64,86,88,89,90]. Table 1 summarizes the nutritional characteristics of insects slaughtered by different treatments.

### 6.1. Protein Quality and Digestibility

Proteins represent the most functionally and nutritionally relevant macronutrient in edible insects. Their digestibility and amino acid availability are highly sensitive to thermal and biochemical stress occurring during slaughter. Thermal methods such as boiling, blanching, or direct drying induce protein denaturation, aggregation, and, in some cases, irreversible crosslinking, which can reduce enzymatic hydrolysis during digestion [12].

While moderate heat treatment can inactivate endogenous proteases and stabilize protein fractions, excessive thermal exposure promotes Maillard reactions, particularly in the presence of reducing sugars, leading to lysine loss and reduced protein quality [91]. In contrast, freezing-based slaughter, especially when followed by gentle drying or lyophilization, preserves native protein structures and results in higher solubility and *in vitro* digestibility [13,71,92,93].

Crude protein content is also influenced by the slaughtering method. For example, the highest crude protein content in *H. illucens* larvae powder was found in asphyxiated larvae vacuum-treated with 52.5% dm, while mechanical, other asphyxiation and heat treatments showed statistically (*p* < 0.001) lower values (39.3–46.8% dm) [71].

Evidence from comparative studies on *H. illucens* larvae and prepupae demonstrates that slaughter conditions modulate protein-related parameters through their effects on enzymatic activity, oxidation, pH, and structural integrity of the protein matrix. Thermal enzyme-inactivating methods, particularly blanching, consistently result in superior protein stability. In *H. illucens* larvae, blanching (typically 40–60 s at ~100 °C) markedly reduced indicators of lipid oxidation and protein-related browning reactions compared to non-thermal or slow-killing methods. Pre-slaughter CO_2_ stunning has been shown to further mitigate stress-induced proteolysis. Leni et al. [14] reported that crickets subjected to CO_2_ prior to thermal processing exhibited improved protein extractability and reduced browning, suggesting that metabolic suppression before death contributes to protein preservation. These findings collectively support the view that slaughter method selection should be considered a pre-analytical control point in insect protein processing, with direct implications for digestibility, extractability, and the design of protein- and peptide-based ingredients.

### 6.2. Lipid Composition and Oxidative Stability

In edible insects, the slaughter method can influence the lipid fraction through two main mechanisms: (i) direct chemical modifications of lipids, including hydrolysis and oxidation, and (ii) indirect effects on apparent lipid composition due to enzymatic inactivation, moisture loss, or co-extraction of minor compounds during processing. These effects are particularly relevant in lipid-rich species such as *H. illucens* and *T. molitor* larvae, where lipids constitute a valuable coproduct but also represent a major source of oxidative instability during processing and storage.

Slaughter methods may influence lipid stability by affecting endogenous enzymatic activity and post-mortem biochemical reactions. Experimental evidence from *Hermetia illucens* prepupae demonstrates that killing conditions can strongly alter lipid class distribution during storage. In a detailed lipidomic study, Caligiani et al. [63] compared freezing and blanching as killing methods and reported that while fatty acid and sterol profiles remained relatively stable, the distribution of lipid classes changed markedly. Prepupae killed by freezing exhibited extensive lipolysis, with free fatty acids increasing dramatically during frozen storage and exceeding 90% of the lipid fraction after two months. In contrast, blanching (100 °C for 40 s) effectively inactivated endogenous lipases and preserved triacylglycerol-rich lipid fractions. These results indicate that slow killing methods, such as freezing, may allow continued enzymatic activity after death, whereas rapid thermal treatments stabilize lipid fractions by inactivating hydrolytic enzymes.

Regarding proximate composition, the impact of slaughter method on total lipid content is often modest and species-dependent. For instance, in *T. molitor* and *Zophobas morio*, comparisons between blanching and freezing revealed only small differences in lipid content (*T. molitor* 28.0 vs. 29.4% lipid, *Zophobas morio* 39.1 vs. 40.1% lipid dry matter basis), indicating that the killing method does not dramatically alter total lipid levels under these conditions [88]. However, even when total lipid content remains stable, fatty acid profiles and derived nutritional indices require careful interpretation. In the same study, fatty acid distributions were largely conserved across slaughter methods, with species-specific patterns dominating lipid composition (*T. molitor*: ΣSFA ≈ 29.6–30.2, ΣMUFA ≈ 46.6–47.3, ΣPUFA ≈ 23.1; *Zophobas morio*: ΣSFA ≈ 45.3–45.5, ΣMUFA ≈ 32.4–32.5, ΣPUFA ≈ 22.1) [88]. Both species also exhibited high n-6/n-3 ratios (>21), indicating a predominance of omega-6 over omega-3 fatty acids, indicating a predominance of omega-6 fatty acids, which may have nutritional implications depending on dietary context. These results suggest that species identity is the primary determinant of lipid quality, while slaughter method may act as a secondary modulator depending on processing context.

The impact of slaughter method on lipid composition and oxidative stability has also been demonstrated in house crickets. Singh et al. [89] reported that non-thermal killing strategies—including freezing, CO_2_ exposure, and vacuum asphyxiation—resulted in higher proportions of polyunsaturated fatty acids (approximately 41–42% of total fatty acids), whereas heat-based treatments such as blanching and steaming reduced PUFA proportions to approximately 37–38%. During refrigerated storage, cricket powders derived from thermal killing methods also exhibited higher lipid oxidation, measured as TBARS after 16 weeks, compared with powders obtained from freezing or CO_2_ treatments. These results suggest that slaughter strategies that avoid intense thermal exposure may better preserve lipid integrity, although they may also influence other quality parameters such as digestibility.

The ether extract (non-polar fraction) of *H. illucens* samples sacrificed by different methods has shown that most slaughtering treatments have no effect on total lipids; the differences in their content were not statistically significant among mechanical treatments, asphyxiation and heating (27.3–29.1% dmb) [71].

In *H. illucens* larvae, lipid content is substantially higher, typically ranging from approximately 35–40% on a dmb. This high lipid load amplifies the technological relevance of even small differences in oxidative status, as minor increases in oxidation can significantly reduce shelf-life and sensory quality [86]. For instance, Larouche et al. [11] reported primary lipid oxidation values of approximately 4.6 mg CHP eq/kg and secondary oxidation values near 1.0 mg MDA/kg following blanching, which were among the lowest across ten evaluated killing methods.

Across studies, a consistent pattern emerges in which slaughter method strongly determines the initial oxidative state of the lipid fraction. In a comprehensive comparison of ten slaughter methods—including blanching, desiccation, freezing at −20/−40 °C, liquid nitrogen, CO_2_/N_2_/vacuum asphyxiation, grinding, and high-pressure processing—primary lipid oxidation (hydroperoxides, FOX assay) was approximately twice as high in larvae subjected to asphyxiation methods (CO_2_/N_2_/vacuum: 18.6–19.4 mg CHP eq/kg) compared to all other treatments (5.8–7.9 mg CHP eq/kg) [11]. This finding indicates that prolonged time to death and stress-associated metabolic activity can predispose lipids to oxidation prior to stabilization.

Secondary oxidation (TBARS) further highlighted the protective effect of rapid enzyme-inactivating treatments. Blanching resulted in the lowest TBARS values (1.0 mg MDA/kg), whereas desiccation produced the highest values (2.5 mg MDA/kg) [11]. These results are consistent with the rapid inactivation of lipolytic and oxidative enzymes by short thermal treatments, although they do not eliminate all oxidative reactions.

An especially informative indicator of lipid stability is fat acidity, which reflects free fatty acid accumulation and susceptibility to oxidation. In *H. illucens* larvae, fat acidity values varied dramatically with slaughter method, ranging from extremely high levels after blending (84.9 mg KOH/100 g fat) to intermediate values following freezing (13.9), CO_2_ (10.4), and vacuum (12.2), and very low levels after blanching (0.77) or CO_2_ + blanching (0.55) [71]. These results demonstrate that methods allowing prolonged enzymatic activity prior to stabilization substantially increase lipid hydrolysis, accelerating oxidative deterioration and off-flavor development. Storage studies further confirmed that TBARS increased more rapidly in lipids obtained from blending, freezing, vacuum, and CO_2_ treatments, whereas blanching and CO_2_ + blanching consistently exhibited lower oxidation rates over 28 days of storage [71].

Importantly, the effects of slaughter methods on lipid oxidation are not isolated but are amplified or mitigated by subsequent drying and defatting operations. In BSFL, blanching (90 °C, 40 s; 1:10 *w*/*v*) and freezing (−20 °C, 24 h) were combined with oven-drying (65 °C, 24 h) or freeze-drying (4 days, −20–20 °C), followed by either mechanical pressing (126–140 °C head temperature) or supercritical CO_2_ extraction (450 bar, 60 °C) [64].

Freeze-killed insects consistently retain more favorable lipid profiles, preserving polyunsaturated fatty acids and minimizing the formation of secondary oxidation products [7,93]. In contrast, direct thermal drying or prolonged heating accelerates lipid peroxidation, particularly in lipid-rich larvae such as *T. molitor* and *H. illucens* [94,95]. Under standard storage conditions, oils derived from freezing generally oxidized more slowly than those obtained after blanching, as reflected by peroxide value (PV) [64]. These findings suggest that while thermal treatments such as blanching may reduce certain oxidation markers through enzyme inactivation, they may also promote the loss of endogenous antioxidants or induce heat-related degradation, thereby affecting long-term stability. Conversely, non-thermal approaches such as freezing and freeze-drying may better preserve antioxidant systems but require strict control to prevent residual enzymatic activity [64].

Boiling presents an intermediate case: while immersion in water limits oxygen exposure and slows oxidation, prolonged treatment can result in lipid losses through leaching. Species-specific lipid composition further modulates these effects; for example, lauric-acid-rich *H. illucens* larvae exhibit greater thermal stability than PUFA-rich cricket species.

Processing conditions applied immediately after insect killing can also influence lipid oxidation during storage. In a comparative study on *T. molitor*, Lenaerts et al. [65] evaluated the effects of microwave drying and freeze drying following a short blanching step used to kill the larvae. Although both drying technologies produced similar proximate compositions, freeze-dried mealworms exhibited markedly higher lipid oxidation levels, with peroxide values reaching approximately 125 mEq O_2_/kg fat compared with values below the detection limit in microwave-dried samples. The authors suggested that the porous structure generated during freeze drying may increase lipid exposure to oxygen, thereby accelerating oxidative reactions. These findings illustrate how post-slaughter processing conditions can substantially influence the oxidative stability of insect-derived lipids.

Collectively, available evidence suggests that slaughter methods may influence the initial biochemical state of lipids for lipid hydrolysis and oxidation. This initial state can be substantially amplified or mitigated by downstream drying and defatting operations. Methods that rapidly inactivate enzymes—such as blanching or CO_2_ pre-stunning followed by blanching—consistently reduce fat acidity, oxidative markers, and microbial loads. However, non-thermal strategies combined with gentle drying and mechanical pressing may better preserve endogenous antioxidants and extend lipid shelf-life.

### 6.3. Vitamin Retention

Edible insects contain a diverse vitamin profile, including B-complex vitamins, tocopherols, and provitamin A carotenoids. Many of these compounds are heat-labile and vulnerable to oxidative stress. Boiling tends to preserve water-soluble vitamins better than dry-heat treatments, albeit with significant leaching losses reported for thiamine and folate [96]. Dry-heat methods such as roasting or oven drying cause substantial degradation of carotenoids and vitamin E, with reported losses exceeding 50% depending on processing conditions. In contrast, freezing and CO_2_-based slaughter reduce pre-mortem metabolic degradation and enzymatic vitamin loss, resulting in higher retention of labile micronutrients [14].

### 6.4. Mineral Content and Bioavailability

Insects are valuable sources of minerals such as iron, zinc, calcium, and phosphorus, though their bioavailability is influenced by matrix interactions with chitin and proteins. Slaughter methods modify these interactions indirectly through protein denaturation and structural disruption. Asphyxiation (with CO_2_ or vacuum) preserves mineral content- or crude ash-effectively (>7.4% dm crude), whereas boiling can enhance mineral extractability at the expense of leaching losses [71]. Mechanical crushing without immediate stabilization may reduce mineral bioavailability by promoting aggregation of chitin–protein–mineral complexes resistant to digestion [97].

### 6.5. Nutritional Trade-Offs and the Slaughter–to–Stabilization Continuum

No single slaughter method optimally preserves all nutritional attributes of edible insects, highlighting the importance of evaluating insect processing within an integrated slaughter–to–stabilization continuum. Different strategies produce distinct trade-offs among microbial safety, nutrient retention, and technological feasibility. For example, freezing is often associated with better preservation of lipids and heat-sensitive compounds but requires substantial energy input; boiling or blanching effectively improves microbial safety yet may degrade thermolabile nutrients; CO_2_ stunning may provide intermediate outcomes; and mechanical killing methods, while economically attractive, may contribute to biochemical degradation if processing conditions are poorly controlled. These contrasts illustrate that slaughter methods cannot be evaluated solely on the basis of a single nutritional parameter. Instead, they should be considered within integrated slaughter–processing strategies that combine pre-stunning, controlled thermal treatments, and appropriate post-slaughter stabilization steps in order to balance nutritional quality, animal welfare considerations, and industrial practicality.

**Figure 2:**
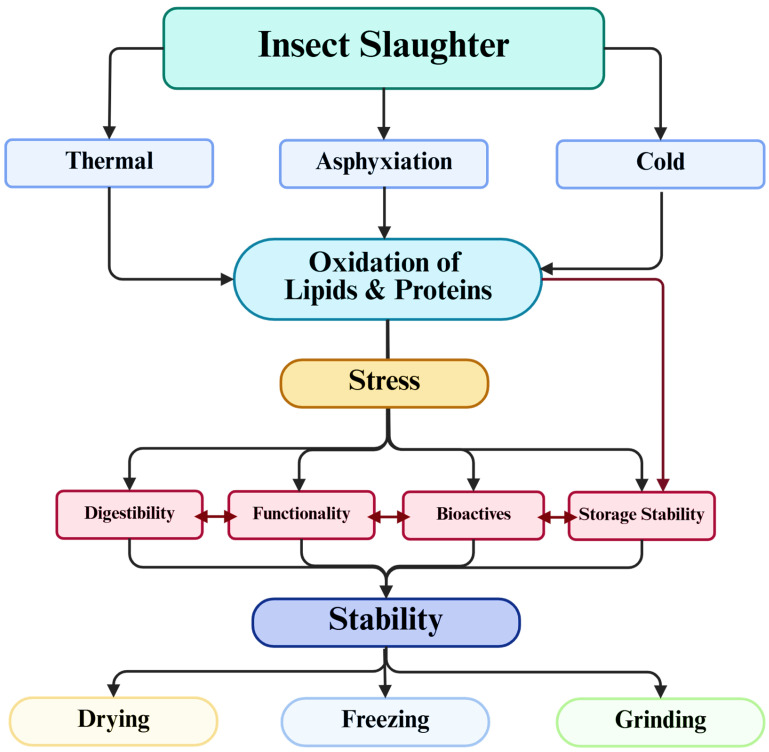
Conceptual framework of the insect slaughter–to–stabilization continuum and its effects on ingredient quality.

This perspective is conceptualized in the slaughter–to–stabilization continuum illustrated in Figure 2. In this framework, slaughter methods—including thermal exposure, asphyxiation, or cold treatments—represent the first critical control point in the processing chain. These procedures can induce physiological stress responses that trigger biochemical processes such as lipid and protein oxidation. The magnitude of these early biochemical changes can subsequently influence several downstream quality attributes of insect-derived ingredients, including protein digestibility, techno-functional properties, preservation of bioactive compounds, and oxidative stability during storage. Importantly, these outcomes are not determined by slaughter conditions alone but emerge from interactions with subsequent stabilization steps such as drying, freezing, or grinding. Together, these operations shape the final physicochemical and nutritional characteristics of insect-based ingredients.

Empirical evidence supports this integrated interpretation of insect processing. For instance, Singh et al. [89] demonstrated that different killing methods applied to *A. domesticus* affected not only immediate physicochemical characteristics but also lipid oxidation dynamics during extended storage. Their results showed that thermal treatments increased protein oxidation and altered fatty acid composition, whereas non-thermal methods preserved polyunsaturated fatty acids but reduced protein digestibility. Such findings illustrate that the choice of slaughter strategy can propagate through subsequent processing stages, influencing both short-term ingredient properties and long-term biochemical stability.

Taken together, these observations reinforce the importance of approaching insect processing from a systems perspective. Rather than treating slaughter as an isolated pre-processing step, it should be understood as the initial stage of a broader slaughter–to–stabilization continuum in which killing methods interact with downstream processing operations to determine the final nutritional, biochemical, and functional performance of insect-derived ingredients.

## 7. Impact on Bioactive Compounds

Beyond basic nutrition, edible insects contain a range of bioactive compounds, including antimicrobial peptides, phenolic compounds, chitin derivatives, and bioactive lipids, which contribute to health-promoting and functional properties [98,99,100,101,102]. These compounds are particularly sensitive to physiological stress and thermal degradation, rendering slaughter methods a decisive factor in their preservation.

Thermal slaughter methods, especially boiling and drying, degrade heat-sensitive bioactives such as phenolics and antimicrobial peptides, leading to reduced antioxidant and antimicrobial capacity [50,93]. Chitin and chitosan structures may also be altered under intense heat, diminishing their prebiotic and immunomodulatory activity [103].

Freezing-based slaughter preserves bioactive integrity by minimizing thermal and oxidative stress. Insects killed by freezing and subsequently dried under controlled conditions retain higher concentrations of phenolics and bioactive lipids compared to those subjected to direct thermal killing [7,93]. CO_2_ further limits stress-induced metabolic pathways that can degrade sensitive bioactives [14].

In *H. illucens* larvae, slaughter strategies that rapidly arrest metabolism while minimizing thermal stress appear to better preserve antioxidant capacity. In particular, freezing-based methods combined with non-thermal drying (freeze-drying) have been associated with enhanced antioxidant preservation, as reflected by a strong inverse relationship between peroxide value (PV) and antioxidant activity measured by the ABTS assay during storage [64]. Oils obtained from freeze-dried larvae slaughtered by freezing and subsequently extracted by mechanical pressing exhibited minimal changes in PV even after six months, suggesting effective retention of antioxidant compounds capable of inhibiting lipid oxidation over extended periods [64].

In contrast, thermal treatments such as blanching, while effective at inactivating oxidative enzymes and reducing immediate lipid oxidation markers (e.g., lower TBARS and fat acidity), may simultaneously reduce the pool of heat-sensitive endogenous antioxidants, thereby accelerating oxidation during long-term storage when combined with high-temperature defatting processes such as supercritical CO_2_ extraction [11]. This trade-off is evident in studies where blanching followed by SFE resulted in more rapid increases in PV compared to freezing-based approaches, with PV exceeding 5 mEq O_2_/kg within approximately two months, whereas freezing-based treatments delayed this threshold to around six months [64]. These findings suggest that antioxidant preservation depends not only on suppressing enzymatic oxidation at slaughter but also on maintaining low thermal and oxidative stress throughout the entire processing chain. Collectively, the data indicates that slaughter methods establishing a milder physicochemical environment—particularly those avoiding prolonged heat exposure—are more likely to conserve endogenous antioxidants, thereby enhancing lipid oxidative stability and extending the shelf-life of insect-derived oils and lipid-containing ingredients.

## 8. Techno-Functional Implications of Slaughter Techniques

In the context of insect-derived ingredients, nutritional and functional quality are closely interconnected. Changes in biochemical composition induced by slaughter and stabilization processes can simultaneously affect nutrient retention, protein digestibility, lipid stability, and techno-functional properties such as emulsification or water-holding capacity. Techno-functional properties determine the suitability of insect-derived ingredients for specific food and feed applications. These properties are strongly influenced by protein structure, lipid integrity, and matrix interactions established at the point of slaughter.

### 8.1. Protein Solubility and Structural Integrity

Protein solubility is essential for applications in beverages, emulsions, and protein isolates [104]. Freezing preserves native protein conformations, resulting in higher solubility and extraction yields [93]. In contrast, boiling and thermal drying induce aggregation and disulfide bond formation, reducing solubility and limiting functionality [12]. CO_2_ stunning prior to heat treatment mitigates stress-induced proteolysis and improves solubility outcomes, as demonstrated in cricket protein extracts [14].

Direct evidence linking slaughter method to protein hydrolysis was provided by [86], who evaluated *in vitro* degree of hydrolysis (DH) in black soldier fly larvae subjected to different killing strategies. Larvae slaughtered by conventional freezing at −18 °C exhibited a significantly lower DH (~19.5%) compared with those subjected to blanching + freezing (~26.9%) or liquid nitrogen freezing (~27.5%). These results indicate that rapid enzyme inactivation (blanching) or ultrafast cooling (liquid nitrogen) yields protein substrates that are more accessible to digestive enzymes. Drying temperature further modulated these outcomes. At a drying temperature of 70 °C, DH values were maximized, whereas drying at 90 °C resulted in significantly lower DH, which the authors attributed to excessive denaturation and aggregation limiting enzyme accessibility [86]. This interaction highlights that the slaughter method and post-mortem thermal exposure act synergistically to determine protein hydrolysis potential.

Supporting these findings, Zhen et al. [71] reported that CO_2_ + blanching treatments produced the highest concentrations of free amino acids in the supernatant following simulated digestion, indicating enhanced release of soluble protein fractions relative to freezing or mechanical blending. Although these values were not expressed as DH percentages, they corroborate the notion that enzyme-inactivating slaughter methods favor peptide liberation. Post-mortem pH is a critical parameter influencing protein solubility and extraction efficiency. Zhen et al. [71] showed that blanching and CO_2_ + blanching significantly increased pH values to approximately 7.31–7.47, compared to ~6.1–6.5 in freezing, vacuum, or CO_2_-only treatments. Elevated pH may reduce protein aggregation near the isoelectric point and enhance solubility during aqueous or alkaline extraction, thereby indirectly improving protein concentration and isolation potential.

Mechanical blending or grinding without immediate stabilization consistently resulted in the poorest protein-related outcomes. Zhen et al. [71] identified blending as the treatment associated with the highest degree of quality deterioration, including increased browning and oxidative instability. From a protein perspective, uncontrolled mechanical disruption likely accelerates the release of endogenous enzymes and pro-oxidants, promoting protein modification and reducing suitability for controlled hydrolysis or functional ingredient production.

Additional evidence supporting the importance of slaughter conditions for protein functionality has been reported for the *A. domesticus*. Singh et al. [89] compared seven killing methods—including blanching (40 s at 100 °C), steaming (2 min 15 s), freezing (−20 °C for 2 h 10 min), CO_2_ exposure (2 h), vacuum, plastic-bag asphyxiation, and CO_2_ followed by blanching—and evaluated their effects on physicochemical traits, nutritional composition, digestibility, and oxidative stability of cricket powder. Their results showed that thermal treatments increased protein oxidation, with CO_2_ + blanching producing the highest carbonyl formation (55.1 nmol mg^−1^ protein), whereas non-thermal methods such as freezing, vacuum, or CO_2_ resulted in lower oxidation levels. However, these same non-thermal methods produced lower *in vitro* protein digestibility compared with heat-based treatments. These results illustrate a clear technological trade-off between oxidative stability and digestibility depending on the killing strategy applied.

Taken together, these studies indicate that slaughter methods that rapidly arrest metabolic and enzymatic activity—such as blanching, CO_2_ + blanching, or liquid nitrogen freezing—may provide advantages over slower methods in several protein-related parameters. Quantitatively, these advantages manifest as 7–8 percentage point increases in DH, reduced browning and oxidation, elevated post-mortem pH, and greater release of soluble amino acids and peptides.

### 8.2. Emulsification and Foaming Properties

Moderate thermal unfolding can enhance emulsification by exposing hydrophobic residues, but excessive denaturation reduces protein flexibility and emulsion stability. Freeze-killed insects generally yield superior emulsifying and foaming capacities due to preserved interfacial activity [105]. Bessa et al. [106] reported that freezing produced an emulsion capacity of ~43% and stability of ~32%, while blanching drastically reduced it to ~10% and ~5%, respectively, on *H. illucens* larvae powder.

### 8.3. Gelation & Water/Oil-Holding Capacity

Gelation properties depend on controlled protein unfolding and network formation. Slaughter methods that induce excessive denaturation result in weak or brittle gels, whereas controlled thermal treatment following freezing or CO_2_ stunning yields improved textural outcomes. Species-specific differences are pronounced; for example, *T. molitor* and *H. illucens* proteins exhibit distinct gelation behaviors under identical conditions [88]. Frozen *H. illucens* larvae (5 days) formed a gel at 5%, while blanching had a negative effect and required 30% to form a gel [106].

Preservation of high-molecular-weight proteins through non-thermal slaughter methods enhances water- (WHC) and oil-holding capacity (OHC) and viscosity, improving yield and mouthfeel in formulated products. WHC and OHC vary with freezing and blanching killing methods on *H. illucens* larvae (WHC 56.27–80.77%; OHC 50.83–68.62%), showing sensitivity of the ingredient to pre-processed conditions [107]. Freezing and blanching on *T. molitor* larvae also showed changes in WHC (in ranges of 1.4–1.79-fold) and OHC (in ranges of 1.2 WAC and OAC 1.5-fold) [108].

### 8.4. Industrial Implications

From an industrial perspective, slaughter methods shape ingredient functionality and application scope. Freeze-killed insects yield high-functionality ingredients suitable for food applications, while heat- or mechanically killed insects are more appropriate for feed or snack products. Understanding these relationships allows manufacturers to tailor slaughter protocols to target markets, improving value chain efficiency.

## 9. Sensory Impact of Slaughter Methods: Texture, Aroma, Flavor, and Color

Sensory attributes play a decisive role in the acceptance and market success of insect-derived foods and feeds. Texture, aroma, flavor, and color are highly sensitive to slaughter-induced biochemical and structural changes, making killing methods a critical upstream determinant of consumer perception.

Thermal slaughter methods such as boiling, blanching, and frying induce pronounced sensory modifications through protein denaturation, lipid oxidation, and Maillard reactions. These processes may generate desirable roasted or nutty flavors in snack-oriented applications but can also introduce bitterness, burnt notes, or off-flavors that limit applicability in neutral or minimally processed foods [12]. Texture-wise, excessive heat often produces firm or rubbery matrices, constraining the use of insect ingredients in emulsified or beverage-type products. Ju et al. [109] compare methods such as blanching, roasting, and freezing on *T. molitor* larvae, and report that blanching and roasting obtained greater overall acceptance (values of 3.33 and 3.53) than freezing (values of 2.00).

Freezing-based slaughter generally preserves lighter coloration, milder aromas, and more neutral flavor profiles when it is used as a drying method [7]. These characteristics are particularly advantageous for Western markets, where consumer acceptance is closely linked to reduced sensory intensity and visual familiarity. CO_2_ stunning prior to thermal processing further reduces stress-related oxidative reactions, resulting in fewer undesirable volatile compounds and improved sensory stability, as observed in cricket-based products [14].

Mechanical crushing and uncontrolled thermal drying often produce the least favorable sensory outcomes. The release of hemolymph, enzymatic browning, and lipid oxidation contribute to dark coloration and muddy or metallic flavors, especially in lipid-rich species [74]. These effects may be acceptable in animal feed applications but significantly limit use in human food products. Importantly, sensory preferences are culturally mediated. In regions with long-standing entomophagy traditions, stronger flavors and darker colors may be acceptable or even desirable, whereas novel markets tend to favor subtle sensory profiles [68]. Slaughter methods should therefore be selected in alignment with target consumer expectations and product formats.

## 10. Toward Ethical, Nutritional, and Functional Standards in Insect Slaughter

The absence of binding, species-specific regulations governing insect slaughter represents a major challenge for the responsible development of the edible insect sector. Taken together, current evidence highlights the need for integrated frameworks that consider welfare outcomes, nutritional quality, and processing efficiency when evaluating insect slaughter methods [110,111,112,113]. Currently, most producers rely on informal practices, personal ethical intuitions, or trial-and-error approaches to killing insects [23,41]. While voluntary guidelines exist, such as those proposed by the International Platform of Insects for Food and Feed (IPIFF), these lack legal enforceability and standardized metrics [114].

Recent welfare-focused analyses further emphasize the need for systematic frameworks to guide humane practices in the rapidly expanding insect farming industry. Barrett et al. [67] proposed the farmed *H. illucens* as a model system for developing welfare standards for insects raised for food and feed. Their work highlights that billions of insects are processed annually and that key practices—including slaughter and depopulation methods—remain poorly standardized across the sector. Developing species-specific welfare indicators and validated slaughter protocols, therefore, represents a critical research priority for the sustainable growth of the industry.

In this context, the precautionary principle provides an important ethical foundation for regulatory development. Behavioral and physiological evidence of nociception in insects, combined with persistent uncertainty regarding subjective experience, supports prioritizing slaughter methods that plausibly minimize potential suffering. This perspective is consistent with broader discussions surrounding the animal sentience precautionary principle and mirrors regulatory trajectories previously observed for cephalopods and decapod crustaceans in Europe [40].

From a scientific standpoint, regulatory frameworks must move beyond binary classifications of “humane” and “inhumane” methods [115]. Instead, standards should incorporate species- and life-stage-specific welfare indicators, including measures of insensibility, time to death, and physiological stress biomarkers. Product quality outcomes—such as nutritional stability or techno-functional performance—may influence processing decisions but represent a distinct dimension from the assessment of slaughter humaneness [116].

Importantly, welfare considerations and product quality are not entirely independent. Slaughter methods that appear to reduce physiological stress may contribute to improved nutritional retention, preservation of bioactive compounds, and techno-functional performance of insect-derived ingredients [14]. However, current evidence remains limited, and further research is required to establish causal relationships between slaughter conditions, physiological stress responses, and ingredient quality.

**Figure 3:**
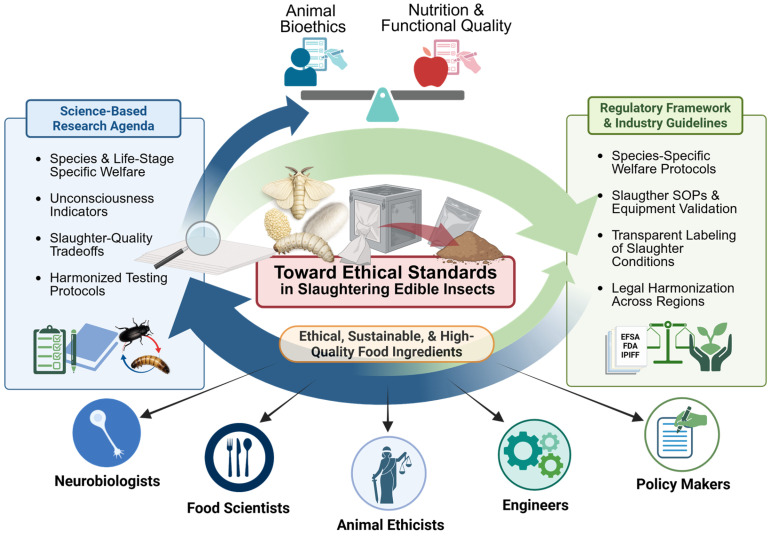
A systems-based framework for establishing ethical standards in edible insect slaughtering.

Advancing ethical and functional insect slaughter will therefore require coordinated research efforts addressing several key gaps (Figure 3). Priority areas include establishing species- and life-stage-specific responses to slaughter methods using behavioral, physiological, and biochemical indicators; defining objective markers of insensibility and distress applicable to insects; evaluating integrated slaughter–processing systems and hybrid approaches (e.g., CO_2_ stunning followed by controlled thermal treatment); and assessing consumer perceptions of welfare-oriented slaughter practices and their influence on market acceptance. Recent optimization studies of mechanical grinding systems illustrate how standardized protocols and equipment design can improve both welfare outcomes and processing consistency (e.g., Barrett et al. [73] and Zacarias et al. [74]).

From a regulatory and industrial perspective, slaughter practices should therefore not be evaluated as isolated interventions but as components of integrated processing systems. Decisions regarding killing methods inevitably interact with subsequent stabilization and processing steps, influencing not only welfare outcomes but also ingredient quality, safety, and economic viability. Future regulatory frameworks should therefore evaluate insect slaughter within the broader slaughter-to-stabilization continuum, recognizing that welfare considerations, processing efficiency, and ingredient quality are closely interconnected within industrial production systems. This perspective aligns with the systems-based framework illustrated in Figure 3, which conceptualizes insect processing as an integrated slaughter-to-stabilization continuum linking welfare science, food technology, and regulatory development.

Another important limitation of the current evidence base concerns the uneven taxonomic and developmental representation of insect species studied in slaughter research. Most available studies focus on a small number of commercially farmed species, particularly *A. domesticus*, *T. molitor*, and *H. illucens*, whereas many other edible insects remain poorly investigated. In addition, research often targets specific life stages such as larvae or adults, leaving other developmental stages comparatively understudied. Expanding research across a broader diversity of insect taxa and life stages will therefore be essential for developing comprehensive and species-appropriate slaughter standards.

Future work should prioritize the development of operational frameworks for evaluating insect slaughter methods. Key research priorities include: (i) identification of reliable welfare indicators (e.g., behavioral responses, neural activity, stress biomarkers); (ii) establishment of physiological markers of insensibility and time-to-death across species and developmental stages; (iii) definition of quality endpoints linking slaughter conditions to nutritional, biochemical, and techno-functional properties; and (iv) development of standardized slaughter protocols tailored to specific species and life stages.

Ultimately, the slaughter of edible insects should be understood as more than a technical pre-processing step. It represents a critical control point shaping ethical legitimacy, product quality, functional performance, and consumer trust. Recognizing insect slaughter as a multidimensional process—integrating insights from bioethics, neurobiology, and food science—offers a pathway toward more responsible and credible entomoculture. Developing scientifically validated and transparent slaughter strategies will be essential for strengthening regulatory frameworks, improving industrial consistency, and supporting the sustainable expansion of insect-based food and feed systems.

## Figures and Tables

**Table 1:** Effect of the method of slaughtering edible insects on nutritional and biochemical properties of derived ingredients.

Species	Slaughter/Pre-Treatment Method	Ingredient Evaluated	Functional Properties Assessed	Main Observed Effect	Ref.
*H. illucens*	Heating by desiccation (air oven, 60 °C, 30 min) and blanching (boiling water, 40 s)	Thawed larvae	Low primary and secondary lipid oxidation	FOX: 5.8–6.4 mg CHP eq/kg wb; TBARS: 1–2.5 mg MDA/kg wb	[11]
*H. illucens*	Freezing at −20 °C, −40 °C and in liquid nitrogen	Thawed larvae	Low secondary lipid oxidation	FOX: 1.7–2.0 mg CHP eq/kg wb; TBARS: 7.2–7.9 mg MDA/kg wb	[11]
*H. illucens*	Asphyxiation with CO_2_ (100%, 120 h, 27 °C), N_2_ (100%, 144 h, 27 °C) and vacuum (120 h, 27 °C)	Thawed larvae	Low secondary lipid oxidation	FOX: 18.6–19.4 mg CHP eq/kg wb; TBARS: 1.6–2.0 mg MDA/kg wb	[11]
*H. illucens*	Mechanical disruption by grinding (2 min, 15,000 rpm) and high hydrostatic pressures (95% vacuum followed by 600 MPa, 3 min at room temp.)	Thawed larvae powder	Low primary and secondary lipid oxidation	FOX: 1.6–2.0 mg CHP eq/kg wb; TBARS: 6.7–7.3 mg MDA/kg wb	[11]
*H. illucens*	Mechanical blending (2 min) and freezing (−20 °C, 6 h)	Larvae powder	High protein and fat content	Crude protein: 39.3–44.9% dm; ether extract: 25.7–27.3% dm; crude ash: 7.3% dm	[54]
*H. illucens*	Asphyxiation by CO_2_ (100%, 120 h, room temp.) and vacuum (120 h)	Larvae powder	High content of nutritional compounds	Crude protein: 46.8–52.5% dm; ether extract: 28.7–29.1% dm; crude ash: 7.4–7.5% dm	[54]
*H. illucens*	Heating by blanching (boiling water, 40 s) and CO_2_ + blanching (100% CO_2_-10 min + boiling water, 40 s)	Larvae powder	High protein and fat content	Crude protein: 46.3–52.5% dm; ether extract: 28.1–28.4% dm; crude ash: 7.0–7.4% dm	[54]
*H. illucens*	Freezing −20 °C	Grinded prepupae	-	Lipolysis > 90% free fatty acids	[63]
*H. illucens*	Blanching 100 °C for 40 s	Grinded prepupae	-	Preserved triacylglycerol-rich lipid fractions by inactivating endogenous lipases	[63]
*Z. morio*	Blanching (100 °C, 40 s)	Defatted-macerated larvae	High lipid diversity and content	Blanching: 39.1% lipid; ΣSFA: 45.5%, ΣMUFA: 32.4%, ΣPUFA: 22.1%, ΣUFA: 54.5%	[69]
*Z. morio*	Freezing (24 h)	Defatted-macerated larvae	High lipid diversity and content	Freezing: 40.1% lipid; ΣSFA: 45.3%, ΣMUFA: 32.5%, ΣPUFA: 22.2%, ΣUFA: 54.7%	[69]
*Z. morio*	Blanching (100 °C, 40 s)	Defatted-macerated larvae	High lipid diversity and content	Blanching: 28% lipid; ΣSFA: 29.6%, ΣMUFA: 47.3%, ΣPUFA: 23.1%, ΣUFA: 70.4%	[69]
*T. molitor*	Freezing (24 h)	Defatted-macerated larvae	High lipid diversity and content	Freezing: 29.4% lipid; ΣSFA: 30.2%, ΣMUFA: 46.6%, ΣPUFA: 23.2%, ΣUFA: 69.8%	[69]
*T. molitor*	Blanching (40 s) followed by microwave or freeze drying	Larvae	-	Freeze-dried larvae showed markedly higher lipid oxidation (125 mEq O_2_/kg fat), whereas microwave drying resulted in minimal oxidation; microwave drying reduced vitamin B12 content	[65]
*T. molitor*	Freezing (−18 °C) + blanching prior to fermentation	Full-fat powder	Foaming and emulsifying properties	Blanching reduced foaming capacity/stability and emulsifying capacity/stability	[89]
*T. molitor*	Freezing + blanching	Full-fat powder	WHC & OHC	WHC and OHC decreased following blanching	[89]
*H. illucens*	Freezing (5 days) vs. blanching (100 °C, 40 s)	Full-fat powder	Gelation capacity	Freezing enabled gel formation at 5% concentration; blanching required 30%	[87]
*H. illucens*	Freezing (5 days) vs. blanching (100 °C, 40 s)	Full-fat powder	Emulsifying capacity and stability	Freezing: ~43% EC, ~32% ES; Blanching: ~10% EC, ~5% ES	[87]
*H. illucens*	Freezing (5 days) vs. blanching (100 °C, 40 s)	Full-fat powder	WHC	WAC ranged from 56.3 to 80.8%, depending on slaughter method	[87]
*H. illucens*	Freezing (5 days) vs. blanching (100 °C, 40 s)	Full-fat powder	OHC	OHC ranged from 50.8 to 68.6%, method-dependent	[87]
*H. illucens*	Freezing (5 days) vs. blanching (100 °C, 40 s)	Full-fat powder	pH	Freezing: ~6.79; Blanching: ~8.94	[87]

CHP, cumene hydroperoxide; EC, emulsion capacity; ES, emulsion stability; FOX, ferrous oxidation-xylenol orange; MDA, malondialdehyde; SFA, saturated fatty acids; MUFA, monounsaturated fatty acids; OHC, oil holding capacity; PUFA, polyunsaturated fatty acids; UFA, unsaturated fatty acids; TBARS, thiobarbituric acid reactive substances; wb, wet basis; WHC, water holding capacity.

## Data Availability

No new data were created or analyzed in this study. Data sharing is not applicable to this article.
